# The Proton Density of States in Confined Water (H_2_O)

**DOI:** 10.3390/ijms20215373

**Published:** 2019-10-29

**Authors:** Sow-Hsin Chen, Carmelo Corsaro, Francesco Mallamace, Enza Fazio, Domenico Mallamace

**Affiliations:** 1 Department of Nuclear Science and Engineering, Massachusetts Institute of Technology (MIT), Cambridge, MA 02139, USA; mallamac@mit.edu; 2 Dipartimento di Scienze Matematiche e Informatiche, Scienze Fisiche e Scienze della Terra (MIFT), Università di Messina, 98166 Messina, Italy; ccorsaro@unime.it (C.C.); enfazio@unime.it (E.F.); mallamaced@unime.it (D.M.); 3 Istituto dei Sistemi Complessi (ISC)-CNR, 00185 Rome, Italy

**Keywords:** water, confined water, density of states, inelastic neutron scattering

## Abstract

The hydrogen density of states (DOS) in confined water has been probed by inelastic neutron scattering spectra in a wide range of its *P*–*T* phase diagram. The liquid–liquid transition and the dynamical crossover from the fragile (super-Arrhenius) to strong (Arrhenius) glass forming behavior have been studied, by taking into account the system polymorphism in both the liquid and amorphous solid phases. The interest is focused in the low energy region of the DOS (E<10 meV) and the data are discussed in terms of the energy landscape (local minima of the potential energy) approach. In this latest research, we consider a unit scale energy (EC) linked to the water local order governed by the hydrogen bonding (HB). All the measured spectra, scaled according to such energy, evidence a universal power law behavior with different exponents (γ) in the strong and fragile glass forming regions, respectively. In the first case, the DOS data obey the Debye squared-frequency law, whereas, in the second one, we obtain a value predicted in terms of the mode-coupling theory (MCT) (γ≃1.6).

## 1. Introduction

The knowledge of a liquid cooled into its amorphous phase still represents for the scientific community an open and not completely clarified problem and the recent studies have led to basic chemico-physical questions that are the focus of a large number of theoretical and experimental studies [1]. All of this beyond the fact that the technological applications of the glasses and the corresponding supercooled liquids are of paramount importance. Because the liquid supercooled below its melting temperature, and its glass phase, is an out of equilibrium (metastable) system, it will be characterized by complex behaviors, in particular in its dynamical properties; a situation evidenced by the thermal behavior of the transport functions such as viscosity, self-diffusion, relaxation times [2] and the excess over the intermolecular density of states (DOS). The physical origin of the so-called fragile-to-strong dynamic crossover (FSDC) remains unclear, although many aspects have been clarified by means of the transport functions. An example of this is the Angell’s classification of supercooled liquids (and glasses) in two categories: strong and fragile [3]. In the first case, the activation energies are well defined between characteristic stages by means of an Arrhenius *T*-dependence (with a single exponential relaxation), whereas the second ones have a multi-relaxation super-Arrhenius behavior described by a stretched exponential form. These special aspects in the liquids behavior on going toward the glass state are accompanied by other phenomena, such as the dynamic heterogeneities (DH) onset [2], the existence (and the role) of a complex energy landscape [4,5,6,7,8] and the FSDC. The FSDC usually takes place at a temperature higher than that of the glass transition ($T_{L}>T_{g}$) inside the supercooled state. This marks relevant features of the dynamic arrested state [7,8,9,10,11,12,13] being accompanied by special events including: the violation of the Stokes–Einstein relation [2], the orientational–translational decoupling [5], the splitting of the relaxation into primary (α) and secondary relaxation processes (β) and the onset of DH [2,14,15,16].

In disordered systems, the study of the vibrational spectrum, being related with the polarizability tensor fluctuations, is traditionally performed by infrared and Raman scattering spectroscopy in the energy range 0<E<500 meV (or frequency ω = 0–100 THz) and the main region of interest for the DOS is 0–100 meV where one can observe the effects directly related with the vibration modes (phonons) like the boson peak (BP) and the librational contribution (whereas the stretching modes are located at the highest *E*). It is well known that neutron scattering is a competitive and more direct technique to study the DOS related with the incoherent self-dynamic structure factor $S_{inc}\left(q,E\right)$ (with *q* and *E* as the scattered wave-vector and energy).

The continuum Debye model, proposed as $g\left(E\right)\propto E^{2}$, describes the behavior in the low-energy region. Supercooled liquids and glasses show excess in such a behavior represented by a collective mode known as the boson peak. The BP appears to be a ubiquity in disordered systems, and many models have tried to interpret its physics although only few have been clarified. Examples of these theories are that of harmonic excitations of a disordered lattice [17] and that of mosaic structures (linked to the DH and the energy landscape) [16]. The findings related with its intensity behavior and energy position are of interest. Similar to the FSDC, the BP appears far from $T_{g}$ and its intensity is proportional to the strong character of liquids: strongest liquids have the most intense BP and vice versa [18,19]. The energy position was instead associated with the lowest frequency at which transverse phonons can propagate in disordered and soft materials [19,20,21]. All of this also reflects the BP dependence on thermodynamic variables such as *T* and density ρ (or pressure *P*). While *T* affects the DOS mainly in the deep supercooled regime near the dynamic arrest, a *P* increase can cause significant BP changes such as a shift toward higher energies and on its shape, height, and width [22]. Other differences may be expected in the BP behavior of strong and fragile glasses. In some molecular liquids [23], the BP has its onset inside the stable liquid phase far from $T_{g}$, an example is water where it is observable above its melting point [24].

An actual datum of glass forming liquids is that both transport functions and the BP can have in the energy landscape frame a common description despite the fact that the underlying time-scales near $T_{g}$ are completely different: i.e., hundreds of seconds in the first case and the picoseconds range for BP. The DOS and thus the BP, being intermolecular processes, are usually studied by means of Raman or inelastic neutron scattering experiments in the energy range 0.2<E<20 meV (ω = 0.02–4 THz) [25,26,27,28,29,30,31]. In specific heat experiments, $C_{p}\left(T\right)$, the BP can be observed in the same systems at low *T*, as an additional contribution to the Einstein–Debye theory prediction for solid of $C_{p}≃T^{3}$.

The aim of the present study is to explore the DOS by means of the incoherent inelastic neutron scattering in confined water and in particular its behaviors as a function of *T* and *P*. Our interest, as discussed in the next section, is focused essentially in the region 0.2<E<10 meV where the DOS is also sensitive to the translational contributions. Water in confined space is a subject of considerable interest for different reasons [32,33,34,35,36]. Firstly, it is due to the role of water in life sciences where it plays a fundamental role in the biological activity starting from the microscopic scales typical of proteins, the cells and membranes and their functions up to the macroscopic one of the living organs, thus involving directly many sciences from biology to chemistry and physics [21,37,38,39]. Secondly, confined water with porous media such as silica gel and zeolites is of special importance for technological applications [40]; other systems that impose spatial constraints to water molecules are polymers, polymer gels, clays, vesicles, microemulsions and micelles where the balance between hydrophilic and hydrophobic originates structural self-organization [25,41,42,43]. However, confined water is also of interest because it can be considered as a model for water inside the deep supercooled regime where bulk water cannot be studied [44].

Liquid water, especially in its supercooled state, presents intriguing and counterintuitive behaviors that have not yet been understood in detail. Beyond this, it is however well established that the hydrogen bond (HB) interactions between water molecules are the key to understand water properties [32,45,46,47,48]. It is noteworthy that HB promotes the formation of a tetrahedrally-coordinated HB network of water that grows in size and stability as the temperature decreases. The HB lifetime increases on decreasing the temperature up to the amorphous phases. A relevant, and well known, characteristic of water is its polymorphism not only in the solid crystal but also in the disordered glass; water in fact possesses a low density amorphous phase (LDA) and a high density amorphous phase (HDA) that form depending on the applied pressure [49]. Starting from the density maximum, this networking is at the origin of many water thermodynamic anomalies and of the apparently diverging behavior of its response functions and transport coefficients observable into the supercooled metastable region. For these reasons, liquid water is the most studied glass forming material [3].

To understand such a special thermodynamics, it has been hypothesized that liquid water can be a mixture of two liquids [37,50,51,52,53]. The *polyamorphism* discovery [49] and the new findings related with the HB networking have confirmed that liquid water is really a mixture (dynamical in character) of two liquids with different densities: a low density (LDL—made by the HB tetrahedral network) and a high density (HDL—monomers, dimers and trimers) liquid, whose predominancy depends on the temperature and pressure [54,55]. At high *T*, the HB lifetime is too small and the water structure is more disordered with respect to low *T* where, instead, the tetrahedral structure (more open and indeed less dense) dominates. With such a reality, and from the observation of a reversible transition from LDA to HDA [49,56], this current study extends the idea of the presence in water of a liquid–liquid phase transition (LLT) and also of a liquid–liquid critical point (LLCP) [57,58].

**Figure 1:**
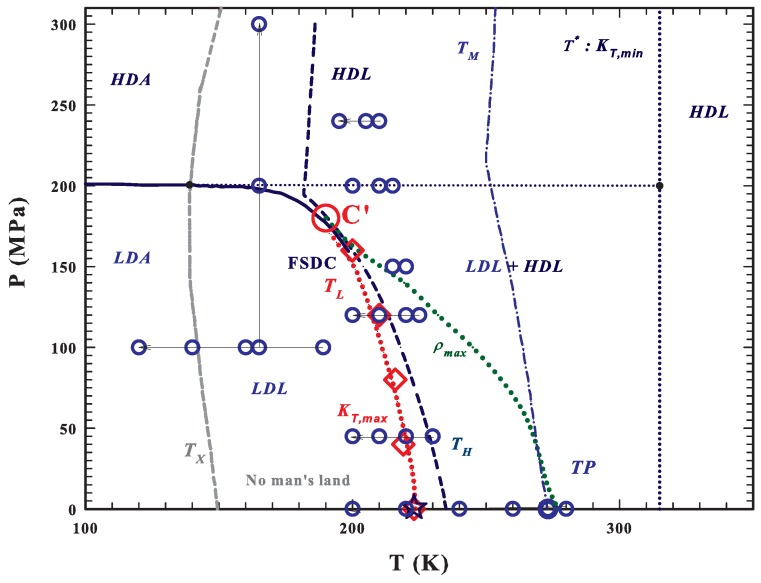
The *T*–*P* water phase diagram [49,54,56]. The metastable supercooled water is located between the homogeneous nucleation $T_{H}$ (dark blue dashed) and the melting $T_{M}$ (blue dotted-dashed) temperature lines. At ambient pressure, below its glass transition $T_{g}\approx 130$ K water is amorphous and on heating crystallizes at $T_{X}\approx 150$ K (dark gray dashed line). The region between $T_{X}$ and $T_{H}$ is the “No-Man’s Land”, where liquid bulk water cannot be studied experimentally. $T_{L}$ (dotted red line) represents the fragile to strong dynamical crossover FSDC line coincident with the Widom line and with the isothermal compressibility, $\kappa_{T}$, maxima ($\kappa_{T}{,}_{\max}$). This latter line ends at the water’s hypothesized second critical point $C^{\prime }$, reported as a large red circle. The FSDC temperatures ($T_{L}$), measured at different pressures using the neutron scattering technique [33], are indicated as red diamonds; these data suggest that $C^{\prime }$ is located at $T_{C\prime }≃200$ K and $P_{C\prime }≃160$ MPa. The line of the density maxima, $\rho_{\max}$, ending at about 180 MPa (green dotted line) and that of the $\kappa_{T}$ minima (blue dotted line), are also reported. The main areas of water polymorphism are indicated as LDA, HDA, LDL, HDL and that of the mixture as LDL+HDL (the only region where liquid water is a fragile former) [59,63]. The large blue circles represent the points of the measured inelastic neutron spectra (INS) [33,59,60,64,66,67].

As shown by the *T*–*P* water phase diagram, see Figure 1, the metastable supercooled water is located between the homogeneous nucleation temperature $T_{H}$ and the melting temperature $T_{M}$ (that at the ambient pressure are 231 and 273 K, respectively). Quenched at temperatures lower than $T_{g}\approx 130$ K, water is amorphous and, if heated, crystallizes into cubic ice at $T_{X}\approx 150$ K. Therefore, in the temperature range between $T_{X}$ and $T_{H}$, known as the “No-Man’s Land”, liquid bulk water cannot be studied experimentally. One method to go inside this region, avoiding crystallization, is to confine water within nanostructures. With such a stratagem it was found, with experiments on the transport functions of confined water, the occurrence of a FSDC at $T_{L}≃223$ K (and 1 bar) as the result of the increase in the HB networking ability of water [59,60]. The FSDC was associated with a domination of LDL over HDL [60], by confirming indeed the existence of the water polymorphism and supporting the scenario of the LLT. It is worth noting that the Stokes–Einstein relation is violated just at $T_{L}$ [60,61]. The FSDC also identifies the LDL-HDL coexistence line, where the thermodynamic response functions of water assume their extremum value (maximum or minimum) [33,62], known as the Widom line [12], whose end point is the water’s hypothesized second critical point $C^{\prime }$. We have to stress here that the dynamical crossover at $T_{L}$ was just explained in terms of the dynamical heterogeneities (or inherent structures) originally proposed just for water [5]. Recently, it has been proposed that there is a temperature line, $T^{*}$, that signs the onset of the water tetrahedral structure [46,63]; as far as such a structure is fully destroyed by the pressure if $P>P^{*}∼$ 200 MPa [64]. Hence, above $T^{*}$, liquid water is made only of HDL, whereas for $T<T^{*}$ is a mixture of HDL and LDL and any temperature decrease means an increase of the LDL fraction. We also must note that $T^{*}$ is the temperature in which, the isobars of the isothermal compressibility $\kappa_{T}$ have its minima and those of the expansivity $\alpha_{P}$ cross in a unique point [46]. Another important observation, made by using NMR and viscosity data, is that $T^{*}$ is also the locus of another FSDC in the sense that for $T>T^{*}$ water rediscovers an Arrhenius behavior so that the pure HDL is a strong liquid.

These confined water studies were the subject of some criticisms based on the idea that for the surface effects (and constraints) confined water is different from the bulk one. However, in a recent study, by using a pulsed-laser-heating technique, the growth rate of crystalline ice, G(T), was measured from 126 to 262 K and from G(T) the self-diffusion of supercooled liquid water has been obtained by using the Wilson–Frenkel model of crystal growth [65]. Such experiment conducted essentially on bulk water (25 molecular monolayers) clearly indicates a fragile-to-strong crossover at ∼233 K and a “strong-to-stronger” crossover at ∼180 K, which fully confirms the corresponding NMR data on confined water [60,61]. Thus, we can stress that such results highlight that confined water can be used to test water dynamics also for $T<<T_{H}$.

Recently, some link between the BP and the Widom line was hypothesized for bulk water by a MD simulation study [13], but experimentally it was observed in confined water [66,67].

The aim of the present work is to study the DOS properties and its relations with the water polymorphism well inside the supercooled liquid regime, and at different pressures, by using inelastic neutron scattering (INS). In detail, we analyzed INS data in silica nanotubes (MCM) confined water. By assuming the central role of the energy landscape and the corresponding inherent structures, with potential minima and saddle points, which determine the physical properties of supercooled systems [7,8], we want to verify these effects in the water DOS; in particular on approaching the FSDC where the system dynamics, on decreasing *T*, it evolves from a super-Arrhenius to a pure Arrhenius temperature dependence. For more clarity, having verified that the energy landscape determines the behavior of the transport functions, here we would like to understand if it plays some role on the local vibrational modes.

In this framework, it has been proposed that the g(E) can give the signature of a phase transition in the space of the stationary points of the energy, from a minima dominated phase (phonon-like region) to a saddle point dominated phase, without phonons [17]. On approaching this phonon-saddle transition, the boson peak goes toward lower frequencies and its intensity shows a critical diverging behavior. In such a frame, the phonon-saddle transition is related to a crossover energy, $E_{C}$, from the phonon dominated scaling $E^{2}$ of g(E) to an $E^{\gamma }$ characterizing the critical region (γ<2). Within this approach, based on the Mode-Coupling Theory (MCT), it is assumed that $E_{C}$ reflects the local molecular order just in terms of an intermolecular distance $\sigma_{0}$ that we assume for water to be the oxygen–oxygen distance $d_{OO}$.

## 2. Experiments

We already reported [33,59,60,64,66,67] numerous DOS data analyses of incoherent inelastic neutron scattering (INS) spectra. The INS experiments here presented were carried out in the following *P*–*T* ranges: 120<T<280 K and 0.1<P<240 MPa (the data are shown in large circles in Figure 1, where the large red circle is the water’s hypothesized second critical point C’, and the red diamonds represent the experimental locations (measured by neutron scattering [33]) of the Widom line reported as a dotted red line). The HRMECS chopper at the Intense Pulsed Neutron Source (IPNS) at the Argonne National Laboratory, was used for P=0.1 MPa; and for the other pressures the Disk Chopper Spectrometer (DCS) at the National Institute of Standards and Technology (NIST) Center for Neutron Research and the Cold Neutron Chopper Spectrometer (CNCS) at the Spallation Neutron Source of the Oak Ridge National Laboratory (ORNL) were used. To study the deep supercooled region, water was confined in a nanoporous silica matrix, MCM-41-S, with 15 Å pore diameter. In these conditions, water remains in the liquid state at temperatures much lower than that of homogeneous nucleation $T_{H}$. Details on the experimental procedure and sample preparation are reported elsewhere [66,67]. The measured energy spectra for a fixed wave-vector q=2 Å${}^{-1}$ (the magnitude of the momentum transfer of the incident neutrons) are indicated as S(q,E). The used wave-vector ensures an energy resolution of about 0.2 meV. In our study, we used the anti-Stokes spectral contributions as in other past works aimed to investigate the BP properties [67]. As it is well known, the neutron spectroscopy probes the molecular motions and the main experimental quantity is the dynamic structure factor S(q,ω) (i.e., the Fourier transform of the van Hove correlation function, G(r,t)) obtained from the measurement of the double differential cross section $d^{2}\sigma /d\Omega d\left(\hbar \omega \right)$, where Ω is the detected solid angle and the transferred energy is E=ℏω (ℏ is the Dirac’s constant).

In this present study, we measured spectra that, for the large incoherent cross section of the hydrogen atoms, are essentially incoherent so that the reported spectra basically correspond to the $S_{inc}\left(q,E\right)$ given by the incoherent (self) double differential cross section:

(1) $$\frac{d^{2}\sigma_{inc}}{d\Omega d(\hbar \omega )}=\frac{N\sigma_{inc}}{4\pi \hbar }{\left(\frac{E_{0}-E}{E_{0}}\right)}^{1/2}S_{inc}\left(q,E\right)$$

where *N* is the number of atoms in the sample, and $\sigma_{inc}=4\pi \left(\bar{b^{2}}-{\bar{b}}^{2}\right)$ is the atomic incoherent scattering cross section (with *b* the corresponding scattering lengths); $E_{0}$ is the incident energy and *E* the energy transfer. For heavy water instead, the measured spectrum contains both coherent and incoherent scattering contributions. It must be noted that such a contribution gives the *q*-dependent density of states (and also the boson peak) of an atom in a liquid or amorphous system as:

(2) $$G_{inc}\left(q,E\right)=\frac{M}{k_{B}T}{\left(\frac{E}{q}\right)}^{2}\exp\left(-\frac{E}{k_{B}T}\right)S_{inc}\left(q,E\right)$$

For an isotropic disordered system, the above function approaches a genuine density of states G(E) as *q*→ 0 (or the reduced density of states $g\left(E\right)=G\left(E\right)/E^{2}$), and for a molecular liquid this limit is practically satisfied for q<3 Å${}^{-1}$. Therefore, a customary way to report the $G_{inc}\left(q,\omega \right)$ is: $G\left(E\right)=\left(E^{2}/q^{2}\right)S_{inc}\left(q,E\right)$.

**Figure 2:**
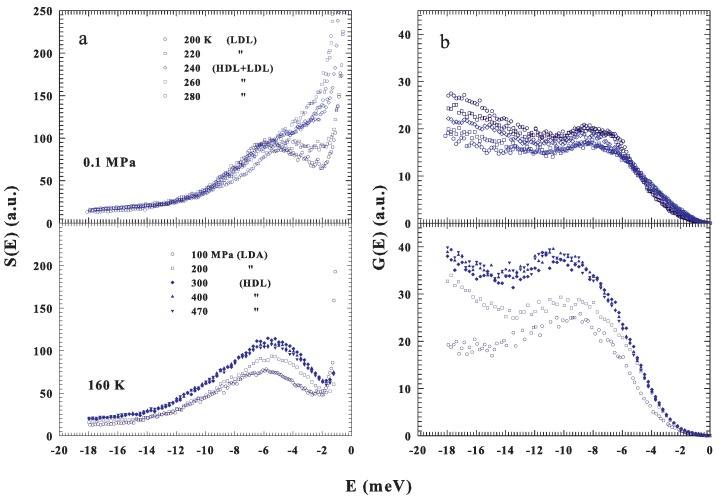
(**a**) Top panel: The INS incoherent spectra S(E) measured at the fixed pressure of 0.1 MPa (T=280,260,240,220 and 200 K). Bottom panel: The S(E) spectra measured at the fixed temperature of 160 K (P=100,200,300,400 and 470 MPa). (**b**) The corresponding intermolecular density of states (DOS).

Figure 2a shows the measured spectra of confined water, S(E), at ambient pressure (0.1 MPa) and different temperatures, T=200, 220, 240, 260 and 280 K (top) and at a fixed temperature T=160 K and different pressures, P=100, 200, 300, 400 and 470 MPa (bottom); the corresponding G(E) are illustrated in Figure 2b. From the spectra one can note the BP presence also under ambient conditions and, how the *P*–*T* changes influence its shapes and energy positions. As proposed in the phase diagram (Figure 1) in the first case, fixed *P*, the *T* changes cross the FSDC at about 223 K and in the second one, fixing *T* and changing *P*, the LLT is crossed at about 200 MPa. The difference between these transitions is that the FSDC is a transition from a region in which the system is a mixture of LDL and HDL (i.e., a fragile region dominated by the energy landscape) to one (strong-Arrhenius) dominated by the LDL. Instead in the second one by increasing the pressure, a transition between the two liquid water phases, LDL to HDL, is induced. All of this marks the evolution and the differences observable in the corresponding G(E).

As can be observed, in both cases, the DOS is influenced by changes in the liquid water thermodynamics. For the LLT, the corresponding *P* effects are more substantial and remarkable than those of *T* at the FSDC. However, for the strong water phase, the G(E) is more intense than that of the fragile and a *P* increase behaves, for its maximum, both a growth and a shift toward the higher energies. All of this well agrees with previous observations in glasses and supercooled liquids [18,19,22], even though our proposed G(E) data deal only with liquid water and its polymorphism. From a simple inspection of the reported water DOS, beside the BP defined from its maxima in intensities and energies, a significant difference emerges in the low energy evolution of G(E), |E|<8 meV, above and below the FSDC (or the LLT), i.e., between the two water liquids that are essentially characterized by a different local order (density). A situation toward which we address our interest just to highlight how the energy landscape approach, used to interpret the physical properties of the water transport functions, can clarify the G(E) thermodynamics.

**Figure 3:**
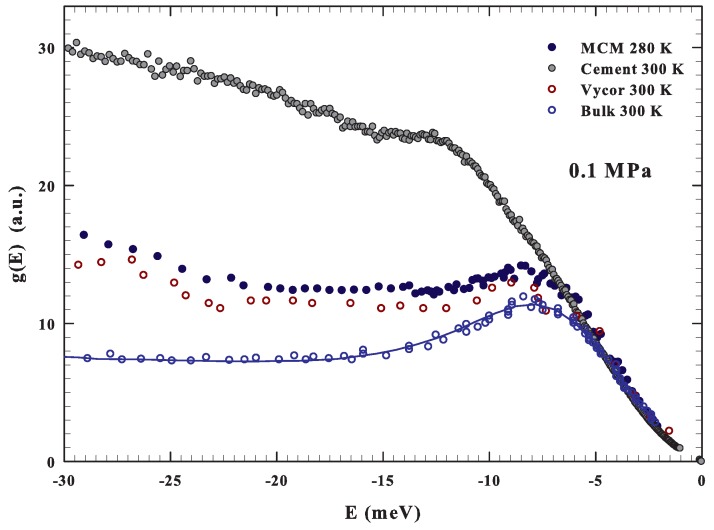
The figure reports, in the energy region −30 to 0 meV, at about the ambient conditions (0.1 MPa and about 300 K) the DOS of bulk [34] and confined water. In the confined case, the G(E) data for water confined in Vycor (300 K) [34], silica MCM nanotubes (280 K) and the cement hydration water (300 K) [69] are reported. For the first three cases, a strong similarity can be observed.

Before starting such a discussion, we show in Figure 3, for the purpose of comparison, the G(E) measured with INS in bulk (T=298 K) [34] and confined water near the room temperature. In the second case, the DOS for water confined in Vycor (pore size 50 Å T=300 K) [34], in MCM (actual data T=280 K) and for the cement hydration one (T=300 K) [68,69] are illustrated. As can be observed, a boson peak is observable at about the same energy for bulk and confined water, although under confinement such a contribution is attenuated.

Here, we notice the clear difference of the DOS of the cement hydration water showing a strong contribution at higher energies. This is presumably due to the hindrance of the proton librational motion caused by the chemical constraints. All of this, just to stress that, as for the self-diffusion [60,65], also in librational modes (|E|<15 meV) there are good similarities between bulk and silica confined water.

## 3. Results and Discussion

The water local properties were the subject of many studies; throughout the years, the water density as far as the related thermodynamical functions (e.g., the isothermal compressibility $\kappa_{T}$ and the expansivity $\alpha_{P}$) have been detailed from both the experimental [46] and theoretical approaches [70,71] in the whole *P*–*T* phase diagram for the amorphous [49,56] and liquid phases [72,73,74,75,76,77,78,79]. Furthermore, by following Bridgmann’s suggestion [72] recently a water minimum has been discovered in confined water (at 0.1 MPa) by means of different experimental approaches [35,36,80]; although the density values are in magnitude about the same, a little difference, depending on the pore sizes, has been observed for temperature of the minimum (some Kelvin). Therefore, the water density ρ(P,T) is well known from 70 to 700 K (i.e., from its amorphous phases to the region of the liquid–vapor critical point) and for 0.1–800 MPa [45,46].

One oxygen originates the water molecules by means of two H–O covalent bonds, by sharing the electron lone pairs (≃4.0 eV binding energy (BE)) and filling the rest two orbits with its nonbonding lone pairs by forming the intermolecular O:H non-covalent van der Waals bond, or the HB (≃0. 1 eV BE). Water thermodynamical properties are thus the effects of its molecular interactions: (i) a noncovalent attractive interaction between two water molecules, the HB, i.e., an electropositive hydrogen atom, on one molecule and an electronegative oxygen atom on another molecule; and (ii) a repulsive intermolecular interaction, the Coulomb repulsion between electron lone-pairs on adjacent oxygen atoms. As a consequence, whereas the HB dominates water in the stable and supercooled regime, the repulsive lone pairs mainly influences the water physics from above the boiling temperature ($T_{b}$) in the sub-critical and critical region (the water critical point CP is located at: $T_{C}=647.1$ K, $P_{C}=22.064$ MPa). To describe the consequent structures, some distances (three) and angles (two) are necessary. Two distances are intermolecular, the oxygen–oxygen $d_{OO}$ and the HB $d_{H}=d_{\left(O:H\right)}$, and one is the covalent intramolecular $d_{L}=d_{\left(O-H\right)}$ that can be assumed as the molecular size. In a vectorial representation, it is: $d_{OO}=d_{H}+d_{L}$. The two angles include the internal θ (the *H*–*O*–*H*) and the intermolecular φ (the HB or H:O–H). The changes in θ are related to the so-called molecular flexibility. Each oxygen atom, according to the Bernal and Fowler ice rule, always tends to have four neighbors by forming a stable tetrahedron (in the higher cubic $C_{3}$ group symmetry), except for water under high temperature and high pressure, also inside the liquid phase [81]. According to these structural considerations, $d_{OO}$ is a quantity directly connected with the density (certainly it evolves as $d_{OO}$ $\approx \rho^{-1/3}$) and from the ρ(P,T) values it can be evaluated; taking in consideration the $C_{3}$ cubic structure, the molecular mass and that at ambient pressure at its density maximum $\rho =1\;{g/\mathrm{cm}}^{3}$, it is $d_{OO}$ $=2.6950\rho^{-1/3}$ [82]. In addition, these interacting water molecules involved in the HB inside the tetrahedron form an asymmetric, coupled, H-bridged oscillator whose relaxations in length and energy (as far as in the local charge distribution) determine the dynamical properties of water in the liquid, amorphous and solid phases, including the vibrational motions.

A common situation experimentally verified is that the BP is an excess in the DOS low frequency region (0.1–10 terahertz) that departs from the Debye law. Originally, some BP interpretations have been proposed on the idea that in such a frequency (or energy) region the phonon dispersion relation is still linear. Instead, in fragile supercooled liquids, such as water, the sound propagation shows marked wave-vector dependence just inside the mesoscopic region (1–10 nm${}^{-1}$) due to the multirelaxing networking structure [8,83]. The explanation of this is in the inherent structures (or energy landscapes) approach. The associated local minima, maxima and saddle points determine the way in which the system vibrational dynamic evolves, up to the transition at the Arrhenius strong phase.That is, similar to the FSDC phenomenon, the system evolves, by decreasing *T*, from a multibasin dynamics with many minima to a molecular trapping in a two steps energy configuration, with a single saddle.

Based on such interpretation, in the past it has been proposed, in terms of the Mode-Coupling Theory (MCT), that the BP is a signature of a phase transition in the energy space from minima (phonon-like region) to a saddle point phase, without phonons; and on approaching this transition it moves toward lower frequencies and the intensity diverges [17]. The transition is also signed by an energy crossover, $E_{BP}$, from the phonon dominated scaling $E^{2}$ of G(E) to an $E^{\gamma }$ characterizing the critical region (MCT gives γ=1.6). Within this MCT approach, the reference energy $E_{C}$ assumes a special role, determining the short scale vibrational excitations, obtained from the local molecular order just in terms of the intermolecular distance $\sigma_{0}=d_{OO}$ as $E_{C}={\left(M\sigma_{0}/Tk_{B}\right)}^{-1/2}$, where *M* denotes the water atomic mass, $k_{B}$ is the Boltzmann constant and $\sigma_{0}$ is obtained from the measured density $\rho =\sigma_{0}^{-3}$.

Our study only deals with a test of the DOS in terms of the energy landscape. Our interest, taking profit from these theoretical suggestions is to give evidence of a different scaling behavior between the fragile and strong phases of liquid water. For such a reason, we measured the DOS in the low energy region at many different *P* and *T* inside the different liquid regions: that of HDL, that of LDL where water must be a strong fluid and that between $T_{L}$ and $T^{*}$ where water is a mixture of both its liquid forms and shows a super-Arrhenius behavior. At the same time, we used separate pathways only to meet the FSDC. In one case, see Figure 1, working at constant pressure, 100 MPa, and changing *T*, we also studied the transition between the LDL and the corresponding amorphous LDA.

**Figure 4:**
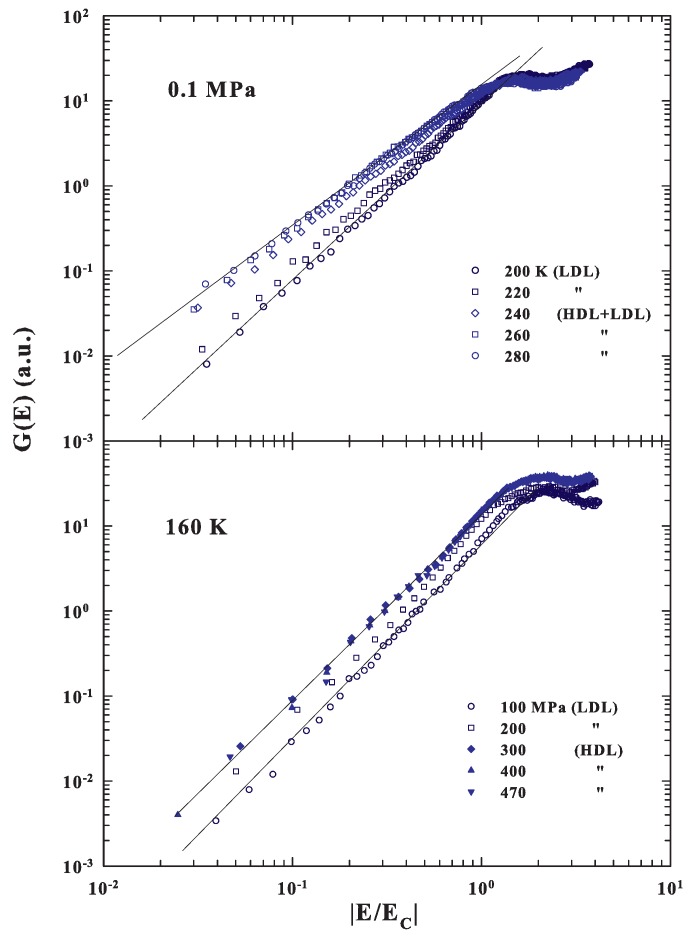
(Top side) A log-log representation of the energy scaled DOS (G(E) vs. $|E/E_{C}|$) obtained from the INS spectra for confined water at the ambient pressure and several temperatures above and below the corresponding temperature of the FSDC (about 223 K). The bottom side instead deals with the same representation of the scaled G(E) for a fixed temperature of 160 K and different pressures in the range 100<P<470 MPa.

Having calculated the energy $E_{C}$ corresponding to the local order by means of the corresponding densities, we evaluated the spectral behavior of the measured DOS after plotting G(E) as a function of the reduced energy $\left|E\right|/E_{C}$. For this, we considered, except for the data at ambient pressure below 240 K, and P=45 MPa and T<230 K where we used the confined water value, only the bulk water densities. Figure 4 (upper side) shows a log-log representation of these energy scaled G(E) in the case of the ambient pressure at several temperatures above and below the corresponding temperature of the FSDC (about 223 K).

As can be observed, the reported G(E) has a different evolution in the fragile region with respect to the strong. In the first case the spectral slope is γ=1.5±0.1, whereas in the strong regime is about 2 (γ=2.05±0.10). Such a result agrees with the cited theoretical suggestions of a MCT criticality in the DOS due to an energy landscape configuration, but also suggests that all of this is a specific property of fragile glasses or liquid glass formers. For strong systems, instead, the low energy behavior of the corresponding G(E) is the one predicted by the continuum Debye model. Certainly these results are strongly coherent with the many experimental observations that water quantities in the supercooled regime, starting from the isothermal compressibility [84] to the other thermodynamical functions [85,86] (including transport [32,45,65]), are characterized by a MCT criticality. In particular, in such a phase, super-Arrhenius temperature behaviors, or in the time scales multirelaxations processes, are observed. The G(E) energy scaling, at the fixed temperature of 160 K, by changing the pressure in a very large interval from 100 to 470 MPa, i.e., by crossing the LLT from LDL to HDL (bottom side of Figure 4), confirms the above results. Here, in fact, the different energy scaled G(E) regards liquid water inside the strong regions, so that in both cases the slopes have about the same Debye behaviors (γ≃2). These findings agree with dielectric experiments, made at the low temperature regime (110<T<150 K), which have shown the strong character of both the pure LDL and HDL phases [87]. To be clear, in the first case, a transition from a fragile phase, dominated by dynamical heterogeneities that imply correlations between time and length scales [2], to a strong phase, stable in elastic mechanical properties and dominated by phonon-like excitation, is observed. In particular, Figure 4 also shows that, for the $E_{C}$ reduced spectra, the energy of the G(E) maxima moves to lower values.

**Figure 5:**
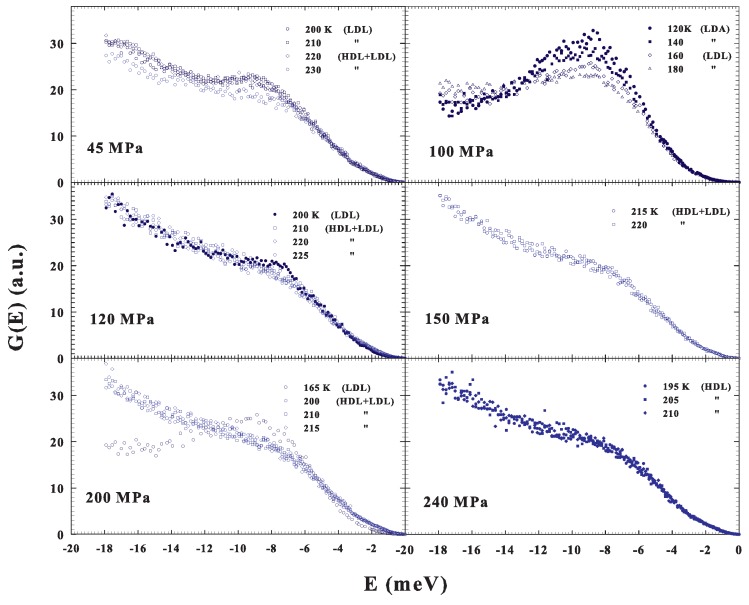
The DOS under different *P*–*T* conditions. For three cases, P=45, 120 and 200 MPa, we observed the water crossover from a fragile to strong liquid (obtained by changing *T*), whereas, in two other cases, P=150 and 240 MPa, we studied, at different *T*, the supercooled liquid inside a precise phase: fragile and strong, respectively. Finally, in one case, P=100 MPa, the data deal with a transition, obtained by decreasing *T*, from the low density liquid to the low density amorphous (LDL to LDA).

As previously said, we also studied the DOS under different *P*–*T* conditions and the resulting G(E) spectra are illustrated in Figure 5. More precisely, in three cases, 45, 120 and 200 MPa, we again reconsidered, by changing *T*, the water crossover from a fragile to strong liquid, and, for 150 and 240 MPa, we studied the supercooled liquid, at different *T*, inside a precise phase: fragile and strong, respectively. Finally, in one case, 100 MPa, the data deal with a transition, obtained by decreasing *T*, from the low density liquid into the low density amorphous (LDL to LDA).

**Figure 6:**
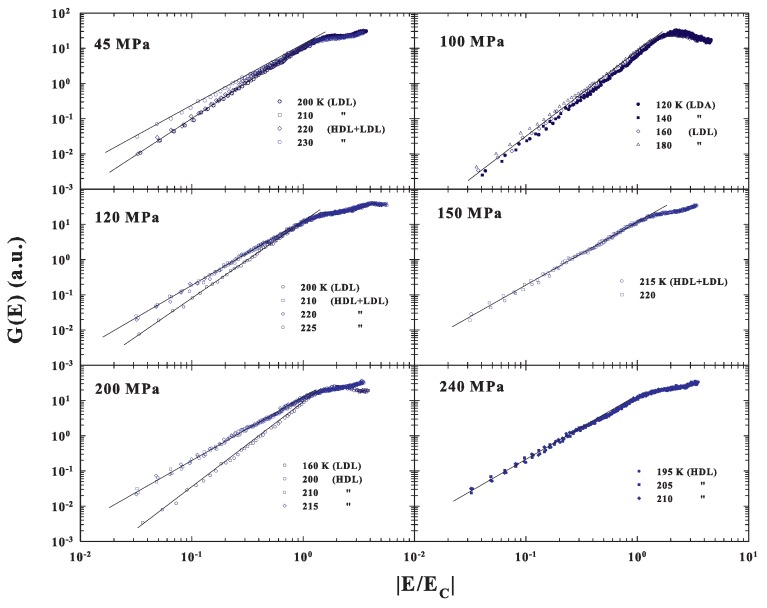
The DOS measured at the different *P* and *T* are reported in a log-log scale as a function of the reduced energy $|E/E_{C}|$. The plot fully confirms the observations of a MCT scaling evolution only when the system is in the fragile, super-Arrhenius, status dominated by the energetic landscape.

Figure 6, where all the corresponding DOS are reported as a function of the reduced energy $\left|E\right|/E_{C}$, fully confirms the previous observations of a MCT scaling evolution only when the system is in the fragile, super-Arrhenius, status dominated by the inherent structures with an energetic landscape configuration. The case of the LDL transition to the LDA measured at 100 MPa is of particular interest. Here, the data behaviors suggest that the amorphous LDA, obtained by decreasing temperature from the corresponding strong glass forming liquid LDL, will maintain the same energetic configuration.

A reason of the observed scaling inside the fragile region lies in the increase of the time scale observed in the supercooled liquid dynamics, as the arrest is approached, which leads to a growing length scale of dynamically correlated regions in the space domain [88], accompanied by a dynamical scaling. This situation is also related with the dynamical decoupling (roto-translational) that takes place just in the saddle region of the energetic landscape [5] implying changes in the system dynamics and also in the associated DOS. It must be stressed that the observed MCT scaling in the LDL + HDL fragile region, is also connected to the incipient growth of the HB lifetime (many orders of magnitude on approaching the FSDC) with an increased stability on the clustering and to a localization of the water dynamics. These tetrahedral HB clusters are dynamical in character, strongly correlated and dependent on the system thermodynamics.

If we go back to the starting considerations that the pure LDL and pure HDL dominated regions are those of a “strong” glass forming liquid, contrary to the mixture HDL + LDL, which is “fragile”, we argue that the obtained findings fully agree with the observed BP and DOS behavior in other glasses and glass forming liquids [2,14,15,16,18,19]: the corresponding spectra are more localized for strong materials rather than broadly distributed for fragile ones.

## 4. Conclusions

We have studied the INS spectra measured for water confined in nanotubes to explore the protons low energy vibrational motions and the boson peak in the *P*–*T* phase diagram from the ambient conditions (T=280 K, P=0.1 MPa) to well inside the supercooled regime and for pressures up to 470 MPa. The main target of the present work was to explore the link between the DOS with the water polymorphism and its liquid–liquid transition that, as evidenced by the recent literature, determines its structural and dynamical properties in the liquid and amorphous phases [55,71]. Our starting point was also based on two considerations: (i) the universality of the fragile-to-strong dynamic crossover (FSDC), characterizing the glass forming supercooled liquid, explained just in terms of the energy landscape [4,5,6,7,8]; and (ii) the observed marked differences in the boson peak between those of strong and fragile glasses and glass-forming systems during a thermodynamic change in their *P*–*T* phase [2,14,15,16,18,19].

These observations have been interpreted in terms of Mode-Coupling Theory (MCT) concepts, for which the DOS is a signature of a phase transition in the energy space from a minima (phonon-like region) to a saddle point phase [17]. Moreover, the transition is evidenced by an energy crossover between two different energy scaling dependent on the local order: an $E^{2}$ (phonon dominated) G(E) scaling and an $E^{\gamma }$ one, characterizing the critical region (MCT gives γ=1.6). In the model, the local order role lies in the energy $E_{C}$, determining the short scale motions, and obtained in terms of the intermolecular distance $\sigma_{0}$, which for water is just the oxygen–oxygen distance $d_{OO}$.

After the $E_{C}$ calculation, at the proper *P*–*T* values, we have scaled all the energies of INS spectra obtaining the evidence of well different behaviors depending if water is in its fragile or strong glass forming energetic configurations. More precisely, in the strong region, the G(E) spectra scale as $E^{2}$, otherwise in the fragile phase the observed scaling is $E^{\gamma }$ with the exponent value of γ=1.6 (i.e., the proposed MCT value [17]). Such a situation holds for liquid and amorphous water in the entire phase diagram.

It must be stressed that the fragile region, being a mixture of the two water liquid forms (LDL plus the HDL), can be assumed, according to such MCT approach, to be a transient critical region, dominated by multirelaxation (super-Arrhenius) processes, where the HB tetrahedral network is not fully developed. In particular, it is a mixture of polydisperse HB clusters and the other water molecules not belonging to these structures, represented by the HDL. The cluster size increases by decreasing *T*, also accompanied by a decreasing in their number distributions as far as the HDL molecules, evolving in the pure LDL phase where the energetic behavior is Arrhenius. A *P* increase behaves oppositely.

New incoherent inelastic neutron scattering and Raman experiments are being planned as a function of *P* and *T*, especially for water contained inside emulsions with a diameter of some microns, a condition where water behaves as in bulk, to study if the DOS intensity shows the predicted MCT critical diverging behavior [17].

## Abbreviations

The following abbreviations are used in this manuscript:

DOS	Density of States
FSDC	Fragile-to-Strong Dynamic Crossover
DH	Dynamic Heterogeneities
BP	Boson peak
